# Baseline Hemostatic Biomarker Assessment Identifies Breast Cancer Patients at High Risk for Venous Thromboembolism During Chemotherapy

**DOI:** 10.3390/cancers17162712

**Published:** 2025-08-20

**Authors:** Marina Marchetti, Patricia Gomez-Rosas, Laura Russo, Carmen Julia Tartari, Silvia Bolognini, Chiara Ticozzi, Debora Romeo, Francesca Schieppati, Luca Barcella, Roberta Sarmiento, Giovanna Masci, Giampietro Gasparini, Filippo De Braud, Carlo Tondini, Armando Santoro, Fausto Petrelli, Francesco Giuliani, Andrea D’Alessio, Roberto Labianca, Anna Falanga

**Affiliations:** 1 Department of Immunohematology and Transfusion Medicine, Hospital Papa Giovanni XXIII, 24127 Bergamo, Italy; patmlhs@hotmail.com (P.G.-R.); annafalanga@yahoo.com (A.F.); 2 School of Medicine and Surgery, University of Milan Bicocca, 20126 Milan, Italy; 3 Department of Biochemistry, Cardiovascular Research Institute Maastricht (CARIM), Maastricht University, 6229 ER Maastricht, The Netherlands; 4 Hematology Unit, Hospital de Oncologia, Unidad Medica de Alta Especialidad (UMAE), Centro Medico Nacional Siglo XXI, Instituto Mexicano del Seguro Social (IMSS), Mexico City 06720, Mexico; 5 Oncology Unit, Hospital San Filippo Neri, 00135 Rome, Italy; 6 Oncology Unit, IRCCS Humanitas Research Hospital, Rozzano, 20089 Milan, Italy; 7 Oncology Unit, IRCCS National Cancer Institute, 20133 Milan, Italy; 8 Oncology Unit, Hospital Papa Giovanni XXIII, 24127 Bergamo, Italy; 9 Oncology Unit, Hospital Treviglio-Caravaggio, 24047 Treviglio, Italy; 10 Oncology Unit, IRCCS Cancer Institute Giovanni Paolo II, 70124 Bari, Italy; 11 Medical Oncology and Internal Medicine, University Vita-Salute San Raffaele, 20132 Milan, Italy; 12 Fondazione ARTET Onlus, 24121 Bergamo, Italy

**Keywords:** metastatic breast cancer, venous thromboembolism, mortality, D-dimer, fibrinogen, hemostatic biomarkers, hypercoagulability, Ki-67

## Abstract

(1) Background: The presence of metastatic disease significantly increases the risk of venous thromboembolism (VTE) in breast cancer, particularly during chemotherapy. Although not categorized as a highly thrombogenic malignancy, the elevated global prevalence of this cancer places a substantial number of patients at risk of thrombosis, which cannot yet be accurately predicted by validated risk assessment models (RAMs), highlighting the need for a dedicated model. (2) Aim: This study aims to develop a RAM for VTE in newly diagnosed metastatic breast cancer patients enrolled in a prospective, observational, and multicenter study. (3) Methods: A cohort of 189 patients beginning antitumor therapy were enrolled and prospectively monitored for VTE and mortality. Blood samples collected at enrollment were tested for D-dimer, fibrinogen, FVIII, prothrombin fragment 1 + 2 (F1 + 2), and thrombin generation (TG). Competing risk analyses were performed to identify significant predictors. (4) Results: Within one year, the cumulative incidences of VTE and mortality were 7.0% and 12%, respectively. Univariable analysis identified high Ki-67, D-dimer, FVIII, fibrinogen, and TG levels, along with low hemoglobin levels, as independent predictors of VTE. Only Ki-67, fibrinogen, FVIII, and hemoglobin were retained as significant predictors in multivariable analysis. These variables were further examined by multiple linear regression, which revealed Ki-67 and fibrinogen as the most significant parameters. A continuous RAM was then developed based on Ki-67 and fibrinogen (c-statistics 0.78), categorizing patients into low-risk and high-risk groups for VTE (2% vs. 13%; SHR 3.6, *p* = 0.018). This stratification could not be achieved using currently validated models for VTE risk. (5) Conclusions: We developed an accurate RAM for VTE that enables the identification of metastatic breast cancer patients at high risk for VTE, which supports clinicians in personalized thromboprophylaxis strategies if externally validated.

## Simple Summary

Breast cancer is classified as a type of malignancy associated with a low relative risk for venous thromboembolism (VTE). Although breast cancer is not usually considered highly thrombogenic, its widespread occurrence worldwide results in a higher rate of VTE in this group compared to the general population. In this study, we aim to assess a prospectively enrolled cohort of metastatic breast cancer patients to ascertain the cumulative incidence of VTE and identify clinical and hemostatic biomarkers that may help to identify patients at greater risk for thrombosis. Our findings indicate that fibrinogen and the oncological proliferation marker Ki-67 are strong predictors of VTE development within one year. Utilizing these biomarkers, we have developed a risk assessment model that can identify patients categorized as high-risk or low-risk for VTE.

## 1. Introduction

Breast cancer is the most common cancer among women worldwide, leading to many cancer-related illnesses and deaths. According to the European Cancer Information System, an estimated 374,800 new cases of breast cancer were diagnosed in women in 2022 [1]. Early diagnosis through educational and prevention efforts has improved treatment success and survival rates [2]. However, about 10% of patients are still diagnosed at advanced stages, with this figure rising to 30% in areas lacking early screening [3,4]. For those diagnosed late, chemotherapy combined with endocrine therapy, immune checkpoint inhibitors (ICI), and targeted treatments remains an effective option [5,6]. The five-year survival rate for human epidermal growth factor receptor 2 (HER-2)-positive and estrogen receptor (ER)-positive molecular subtypes is around 85%, whereas higher levels of the Ki-67, a measure of cellular proliferation, are related to lower survival [7,8]. In addition, breast cancer ranks third in cases of cancer-associated thrombosis [9,10,11]. It is also the type of cancer in which the thrombogenic effect of chemotherapy was first recognized, as reported by Levine et al. as early as 1988 [12].

The mechanism behind venous thromboembolism (VTE) in cancer patients is complex and involves a combination of tumor biology, patient characteristics, and treatment-related factors. Cancer itself increases the risk of thrombosis due to the release of procoagulant factors by tumor cells, activation of the coagulation cascade, and inflammatory responses [13,14]. Furthermore, certain chemotherapeutic agents and targeted therapies may heighten the risk of thrombosis by causing endothelial-vessel damage, platelet activation, and blood flow impairment (Supplementary Table S1) [15,16,17,18].

The occurrence of VTE in patients with metastatic breast cancer who are starting anticancer treatment significantly impacts their disease burden, quality of life, and healthcare costs [9]. VTE is a serious complication that can lead to increased hospitalizations, the need for anticoagulant therapy, and potential interruptions in cancer treatment, all of which can affect overall survival and patient outcomes [19,20]. Given the significant impact of VTE in this cancer population, it is important to provide thromboprophylaxis. However, due to the varying risk of VTE and the potential for bleeding complications from anticoagulants, a universal pharmacological thromboprophylaxis approach is not justified. Current guidelines recommend administering primary pharmacological prophylaxis only to subjects at high risk of thrombosis, while avoiding it in low-risk individuals [21,22,23]. To achieve this goal, they recommend using validated risk assessment models (RAMs), such as the Khorana risk score (KRS), the CATS nomogram, and the COMPASS-CAT score [24,25,26]. However, these RAMs have limitations in their application to outpatient settings and may not adequately capture the changing risk profiles of patients undergoing cancer treatment [27,28].

This highlights the ongoing need for research to develop and validate more effective RAMs for breast cancer patients who are at high risk of VTE. Such models would allow for the implementation of appropriate thromboprophylaxis to reduce the risk of VTE and its complications.

In this context, this multicenter, prospective, observational study on newly diagnosed metastatic breast cancer patients beginning chemotherapy aims to address this gap by evaluating the incidence of VTE and the role of hypercoagulation biomarkers in predicting VTE and mortality within a 12-month follow-up period. Additionally, the study aims to develop a RAM to identify patients who are at an increased risk of VTE.

## 2. Materials and Methods

### 2.1. Study Design and Population

This prospective, observational, multicenter cohort study represents a sub-cohort of the HYPERCAN study (ClinicalTrials.gov ID#NCT02622815). The study enrolled newly diagnosed metastatic breast cancer outpatients, classified by the tumor, node, metastasis (TNM) staging system as TxNxM1, from April 2012 to the end of August 2020, who were candidates for chemotherapy. The study design is described in detail previously [29,30]. Briefly, the inclusion criteria were adult patients (aged ≥ 18 years) with a newly histologically confirmed diagnosis of metastatic breast cancer. Patients were excluded if they had a history of VTE within the past six months, were on anticoagulant therapy at therapeutic doses, or had other significant comorbidities that could confound the study results (i.e., recent acute infection or hospitalization). According to the study protocol, patients were followed up for at least 5 years after enrollment. Clinical data were gathered, including patient demographics, tumor characteristics, treatment regimens, and comorbidities.

The study was conducted following the Declaration of Helsinki and was approved by the institutional review board of the participating center. Written informed consent was obtained from all participants before enrollment.

### 2.2. Blood Collection and Plasma Preparation

Peripheral venous blood was collected before the initiation of any anticancer treatment, in 6 mL Vacutainer tubes containing 0.109 M sodium citrate at a 9:1 *v/v* ratio (Becton Dickinson, Vacutainer, Plymouth, UK). Within two hours of collection, free-platelet plasma was obtained through a two-step centrifugation at 3000× *g* for 10 min at 22 °C. Subsequently, the plasma was stored at liquid nitrogen at −196 °C until its use, following previously established protocols [30]. Blood samples were processed and analyzed at the Immunohematology and Transfusion Medicine Laboratory at Hospital Papa Giovanni XIII in Bergamo, Italy.

### 2.3. Biomarker Assessment

Plasma D-dimer levels were measured on the STA Compact Max 3 coagulation analyzer using the STA Liatest D-Di PLUS; while plasma levels of fibrinogen and factor VIII coagulant activity were measured on the automated coagulometer analyzer ACL TOP500 (Werfen Group, Milan, Italy), with the quantitative fibrinogen assay (QFA) thrombin test (Werfen) and the HemosIL FVIII:c test (Werfen).

Prothrombin fragment 1 + 2 (F1 + 2) levels were determined using an ELISA assay with a commercially available kit (Enzygnost^®^, Siemens Healthcare Diagnostics, Munich, Germany).

Thrombin generation (TG) was performed in duplicate using free-platelet plasma samples by the calibrated automated thrombogram method (CAT assay, Stago, Maastricht, The Netherlands) [31]. The assay was conducted with 5 pM Tissue Factor (TF) and 4 µM phospholipids. The following parameters were obtained from the TG curve: lag time (in minutes, which measures the time from test initiation to signal detection), time to peak (ttP, in minutes, which indicates how long it takes for thrombin concentration to reach its maximum value), peak height (in nM, representing the maximal thrombin concentration), and endogenous thrombin potential (ETP, in nM*min, which is the area under the thrombin time concentration curve) [32].

Assays were performed in strict accordance with the manufacturer’s instructions. The accuracy and reliability of the results were ensured through comprehensive quality control measures [32,33]. These included the integration of internal quality controls (at normal and low analyte levels) supplied by the manufacturer and a third-party plasma sample within each analytical run. For the D-dimer, fibrinogen, and FVIII assays, participation in an External Quality Assessment (EQA) program was also maintained.

### 2.4. Study Outcomes

The primary outcome of this analysis is the occurrence of a symptomatic or incidental VTE within 12 months of starting antitumor treatment. These events must be objectively confirmed through duplex sonography, phlebography, computed tomography, or a ventilation–perfusion lung scan. VTE includes symptomatic deep vein thrombosis (DVT), symptomatic non-fatal pulmonary embolism (PE), fatal PE, incidental proximal DVT, and incidental proximal PE (in segmental arteries or larger). Events were also identified as catheter-related or non-catheter-related thrombosis [34]. Mortality at 12 months from enrollment was considered a secondary outcome. Only events confirmed and validated by the Independent Central Adjudication Committee are included in this analysis.

### 2.5. Statistical Analysis

Categorical variables were reported as frequencies and proportions, while continuous variables were reported as the median and interquartile range (IQR). The chi-square or the Fisher Exact test was used to compare categorical variables. Differences between groups for normally and non-normally distributed quantitative data were tested using the unpaired Student’s *t*-test and the Mann–Whitney U test. Significant associations between variables were tested by Pearson correlation coefficient and plotted by heat map (heatplot; STATA). Univariable and multivariable analyses were conducted using the Fine and Gray proportional hazards regression model with all-cause mortality as the competing risk (FGM, CR methodology, stcrreg STATA) to identify statistically significant prognostic factors for VTE and to avoid overestimating cumulative risks in the presence of substantial underlying mortality, with results expressed as the subdistribution hazard ratio (SHR). Furthermore, the significant (*p* < 0.05) clinical and biomarker predictors of VTE identified by the univariable competitive FGM were included in the multivariable FGM using a forward variable selection algorithm. After obtaining the predictors through multivariable analysis, the independent variables were weighed to identify the most significant ones using a likelihood forward approach with multiple linear regression. A condition index test was applied to exclude multicollinearity among the obtained variables. To construct a RAM, the variables were multiplied by their beta coefficients and then summed to yield a value that was further analyzed using Youden’s index to establish risk categories for VTE. The predictive accuracy of the model was assessed using C-statistics and plotted by the receiver operating characteristic (ROC) curve. A calibration plot was also applied to evaluate the model’s performance, in accordance with TRIPOD guidelines. In addition, FGM evaluated the scores of the published RAMs KRS [25], CATS nomogram [26], COMPASS-CAT [24], and HYPERCAN-VTE [32] to assess their predictive role for 12-month VTE. Univariable Cox proportional hazard regression (HR) models (stcox STATA) were employed to evaluate the predictive value of clinical and laboratory variables concerning the endpoint of mortality. The Kaplan–Meier method was used to estimate survival functions, using the day of study enrollment as the baseline time. The results were compared using the log-rank test. Statistical analyses were conducted using StataCorp. 2019. Stata Statistical Software: Release 16 (StataCorp LLC., College Station, TX, USA).

## 3. Results

### 3.1. Baseline Characteristics of the Study Population

Table 1 shows the characteristics of the study cohort. A total of 189 metastatic breast cancer patients (98% female) with a mean age of 60 years (range, 32–91) were analyzed. ECOG performance was predominantly between 0 and 1 in 87% of the patients, and 9% had an ECOG score of 2. Approximately 54% of the population had at least one comorbidity, with hypertension being the most prevalent. At the time of enrollment, four patients were receiving prophylactic low-molecular-weight heparin (LMWH) and were subsequently excluded from further analysis. Ductal carcinoma was diagnosed in 83% of patients. The most represented molecular subtypes were Luminal B HER2-negative (37%), Luminal B HER2-positive (27%), Luminal A (14%), triple-negative (TN, 11%), and HER2-positive (9%). Chemotherapy was primarily administered as palliative care, with only 3% of patients receiving treatment aimed at achieving a cure, predominantly taxane-based drugs at 62%. Immunotherapy was used in 43% of the patients, and tamoxifen in 53%.

### 3.2. Primary and Secondary Outcomes

Within one year of enrollment, 14 patients experienced VTE in a median time of 173 days (IQR 52–258). In particular, we registered 7 DVT, 5 PE (1 fatal), and 2 DVT plus PE, resulting in a cumulative VTE incidence of 7.5% (95% CI 4–12). No bleeding events were observed during follow-up. According to baseline characteristics, only active or former smokers were significantly more predominant in patients who developed VTE within 12 months compared to those without VTE during follow-up (Table 1). During the same follow-up period, the cumulative incidence of mortality was 12% (95% CI 7–17), with a median time to death of 212 days (IQR 106–306). Mortality was mainly caused by disease progression, except for one death due to a fatal PE.

### 3.3. Hemostatic Biomarkers and Thrombin Generation in Relation to VTE

**Figure 1:**
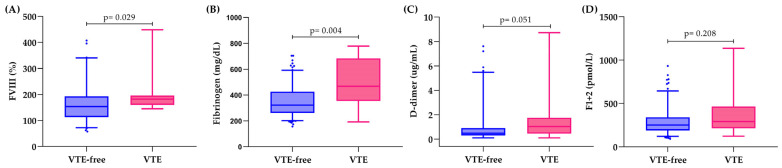
Distribution of hemostatic biomarkers according to the development of VTE within 1 year, and VTE-free. (**A**) Factor VIII; (**B**) fibrinogen; (**C**) D-dimer; (**D**) prothrombin fragment 1 + 2 (F1 + 2). In the box plots, the median is shown by the line inside the box, and the whiskers indicate the interquartile range of the values; dots represent values over the 95th percentile.

Figure 1 shows the baseline levels of biomarkers in patients who experienced VTE and those who remained VTE-free. Patients with 12-month VTE exhibited significantly (*p* < 0.05) higher baseline levels of FVIII and fibrinogen than those who remained VTE-free during the same follow-up period (*p* < 0.05). D-dimer levels were higher in patients who developed VTE compared to VTE-free patients; however, the values did not reach the limit of significance. Specifically, patients with and without 1-year VTE showed median fibrinogen levels of 468 mg/dL (IQR 354–684) and 322 mg/dL (IQR 262–426), respectively. The median FVIII levels were 182% (IQR, 160–196) in patients with VTE and 153% (IQR, 113–193) in those without VTE. D-dimer median levels were 1.04 µg/mL (IQR 0.46–1.75) for VTE patients and 0.49 µg/mL (IQR 0.31–0.91) for VTE-free patients. No significant differences were observed in F1 + 2 levels between the two groups (292 vs. 253 pmol/L).

**Figure 2:**
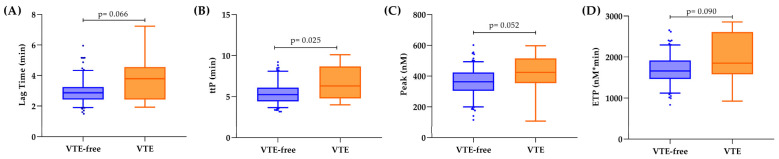
Distribution of thrombin generation parameters according to 1-year VTE and VTE-free patients. (**A**) Lag time; (**B**) time to peak; (**C**) peak of thrombin generation; (**D**) endogenous thrombin potential. In the box plots, the median is shown by the line inside the box, and the whiskers indicate the interquartile range of the values; dots represent values over the 95th percentile.

Figure 2 presents the results of TG. Patients with 12-month VTE had significantly (*p* = 0.025) prolonged ttP compared to patients who remained VTE-free (6.29 min IQR [4.79–8.68] vs. 5.24 min [IQR 4.43–6.09]). Lag time was also prolonged in VTE patients (3.8 vs. 2.9 min), together with higher peak (424 vs. 364 nM) and ETP (1851 vs. 1671 nM*min) of TG values, compared to patients without VTE. However, these values did not achieve statistical significance (*p* > 0.05).

Correlation analysis demonstrated significant associations between all the parameters of TG and the levels of fibrinogen. These associations were stronger in patients who experienced VTE during follow-up compared to those who were VTE-free, as shown in Supplementary Figure S1. Furthermore, in patients with VTE, a strong correlation was observed between F1 + 2 and FVIII levels.

### 3.4. Predictors of 12-Month VTE

Clinical and tumor characteristics and biomarker measurements were evaluated using Fine and Gray univariable analysis, with all-cause death as a competing risk (Table 2). According to this analysis, lower hemoglobin levels (HR, SHR 0.784, *p* = 0.033) and higher proliferation status, as measured by Ki-67 % (SHR 1.021, *p* = 0.046), were independent predictive factors for the development of VTE. In addition, D-dimer, fibrinogen, FVIII, lag time, ttP, and ETP of TG emerged as significant (*p* < 0.05) independent risk factors for 12-month VTE by the FGM univariable analysis.

### 3.5. Development of a Risk Assessment Model

A multivariable analysis approach, considering all the significant variables identified by the FGM univariable analysis, was performed, and FVIII (SHR 1.008, *p* = 0.034), fibrinogen (SHR 1.001, *p* = 0.048), and Ki-67 (SHR 1.055, *p* = 0.017) remained independent risk factors for VTE (Table 2). These variables were then analyzed using linear regression to determine their significance. After forward selection, fibrinogen (β 0.298, *p* < 0.001) and ki-67 (β 0.189, *p* 0.016) remained as the strongest predictors of VTE. The condition index of fibrinogen and Ki-67 was 7.684, thus ruling out multicollinearity. A continuous risk score was developed based on the two variables using the β coefficients from the linear regression. According to the Youden index, a cut-off point was established for stratifying risks.

**Figure 3:**
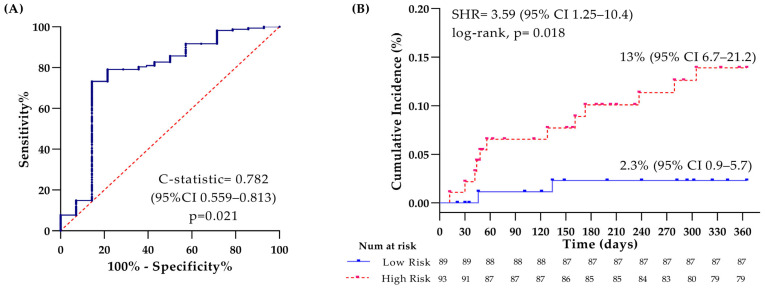
Receiver operating characteristic (ROC) curve of the model and cumulative incidence of venous thromboembolism (VTE) within 1 year. (**A**) Accuracy of the model by ROC curve; (**B**) Kaplan–Meier of the cumulative incidence of VTE at 1-year of follow-up. The dotted red line represents the high-risk group, and the blue line represents the low-risk group.

The model’s accuracy, as indicated by the ROC curve, and the VTE incidence according to high and low risks are shown in Figure 3. A cumulative incidence of 13% (95% CI 7–21) was achieved in the high-risk category compared to 2% (95% CI 0.9–6) in the low-risk group, with SHR 3.59 (95% CI 1.25–10.4), log-rank, *p* = 0.018, c-statistics 0.782, and *p* = 0.021. The calibration of the model demonstrated agreement between the predicted and the observed events at 3 percentiles, as shown in Supplementary Figure S2.

Published VTE RAMs were applied to our cohort, including KRS, COMPASS-CAT, HYPERCAN, and the CATS nomogram, as shown in Supplementary Figure S3. Specifically, KRS, at the original cut-off of 3 points, significantly distinguished between low-risk patients (with a VTE incidence of 5.3%) and intermediate-risk patients (with a VTE rate of 16.3%), with a statistical significance of *p* = 0.015. However, this scoring system could not identify patients at high risk (with a VTE rate of 0%). When KRS was adjusted to a cut-off of 2 points, the VTE rate in the low–intermediate-risk group was 8%, compared to 25% in the high-risk group, though this did not reach statistical significance (log-rank = 0.188). The HYPERCAN and CATS nomograms yielded similar results, with log-rank values of *p* = 0.215 and *p* = 0.240, respectively. In contrast, COMPASS-CAT achieved better stratification, with a low-risk VTE rate of 0% versus 9% in the high-risk group, although it was not statistically significant (log-rank, *p* = 0.079). However, when the COMPASS-CAT score was applied using a cut-off of 11 points, it significantly stratified patients into low-risk (0%) and high-risk (12%) categories, with a significant log-rank value of *p* = 0.045. The accuracy of these RAMS was also assessed using ROC curves, with c-statistics of 0.630 (*p* = 0.106) for the 3-point KRS and 0.527 (*p* = 0.742) for the 2-point KRS. The HYPERCAN score showed a c-statistics of 0.640 (*p* = 0.082), CATS nomogram 0.547 (*p* = 0.560), the original COMPASS-CAT scored 0.595 (*p* = 0.241), and COMPASS-CAT at an 11-point cut-off was 0.616 (*p* = 0.151).

### 3.6. Predictors of 12-Month Mortality

To determine the clinical and laboratory predictors of 12-month mortality, we performed a Cox regression analysis on the baseline characteristics of the cohort, as illustrated in Table 3. Concerning the clinical characteristics, elevated leukocyte levels, lower progesterone receptor expression, an ECOG performance status ≥ 2, and the TN molecular subtype were identified as predictors of one-year mortality. In contrast, treatment with targeted therapy, tamoxifen, and aromatase inhibitors acted as protective factors against mortality at the same follow-up period. Among the hemostatic biomarkers, high baseline levels of D-dimer (HR 1.257, *p* = 0.008), fibrinogen (HR 1.003, *p* = 0.009), and FVIII (HR 1.008, *p* < 0.001) were identified as independent predictors of one-year mortality. After a forward selection of significant predictors from the univariable analysis, the multivariable analysis identified the TN subtype (HR 3.988, *p* < 0.001), elevated leukocytes (HR 1.150, *p* = 0.050), and FVIII levels (HR 1.010, *p* < 0.001) as the strongest predictors of mortality. In addition, patients who developed a 6-month VTE were 3-fold more prone to death within one year of follow-up (HR 3.028, 1.518–6.042; *p* = 0.002).

## 4. Discussion

Breast cancer remains a widely prevalent tumor condition, placing a substantial number of patients at risk for VTE, particularly during chemotherapy [12]. Most validated RAMs often lack accuracy when applied to breast cancer patients, thereby failing to effectively identify individuals who are at elevated risk for VTE [28]. Therefore, our study in a prospective observational cohort of patients with metastatic breast cancer evaluated various clinical and laboratory parameters in relation to VTE to address this critical challenge. The results demonstrated a cumulative incidence of first VTE at 7.5% after one year of follow-up from the initiation of systemic antitumor therapy. A substantial proportion of these events, specifically 79%, occurred within the initial six months, with a median time to VTE occurrence documented at 172 days.

Our findings on VTE rates align with those reported by other prospective and epidemiological studies in breast cancer [9,19,35,36], while being higher than the 1.0% incidence of VTE observed in extensive mixed-cancer epidemiological studies [37]. These differences emphasize the importance of cohort specificity and research design in understanding VTE risk.

To identify potential predictors, we first compared patients who developed VTE with those who remained VTE-free during follow-up. According to this comparison, analysis on hemostatic biomarkers showed that patients who experienced VTE exhibited significantly higher baseline levels of FVIII, fibrinogen, and D-dimer, along with prolonged ttP and a higher peak of TG. Some of these findings have been previously documented in breast cancer, indicating that, despite being classified as a low-VTE risk cancer type, there is a persistent hypercoagulable state that eventually worsens during chemotherapy [35,36]. Regarding factor VIII, two observational prospective studies found that elevated levels of this factor are associated with an increased risk of VTE in cancer [38,39]. In the study by Tafur A. et al., which included 21% of participants with breast cancer at various stages of the disease, it was found that patients with high FVIII levels had a 2.5-fold increased risk of developing VTE during follow-up [40], while in the study by Vormittag R. et al., which included 16% of breast cancer patients, high FVIII levels doubled the risk of VTE [39]. Similar to FVIII, elevation of fibrinogen and D-dimer has also been documented in breast cancer with VTE. A study conducted by Kirwan et al. established that pre-chemotherapy levels of fibrinogen and D-dimer serve as significant predictors for the development of VTE. Specifically, a 1 g increase in fibrinogen was associated with a doubled risk of VTE, while a rise of 1000 ng/mL in D-dimer correlates with a 1.8-fold increased risk [36]. Fibrinogen levels were also evaluated in a prospective study that examined coagulation biomarkers in breast cancer patients who underwent radical mastectomy. The findings revealed no statistically significant difference in fibrinogen levels between patients who developed VTE after surgery and the control group. In contrast, other fibrinogen degradation products (FDP) and D-dimer were identified as predictors for VTE [41]. The increased global capacity to generate thrombin in response to stimuli integrates our recent findings on elevated circulating levels of hypercoagulation biomarkers in this population, indicating an ongoing thrombogenic state, as well as increased fibrin formation and degradation (as evidenced by elevated D-dimer levels). Finally, some TG parameters (i.e., lag-time, ttP, and ETP) were further identified as predictors of VTE. TG potential has been assessed within mixed cancer populations, yielding positive results concerning its predictive value for VTE [42] and other clinical outcomes [30,43]. Indeed, our research group evaluated the role of TG in patients with high-risk breast cancer following extensive surgical resection, raising ETP as a reliable biomarker for cancer prognosis, specifically for early disease recurrence [43]. These findings were subsequently validated through a fully automated platform [44]. It is interesting to note that in the presence of high ETP and peak TG, a short lag time and time to peak are typically expected; however, this is not observed in our patients. As described in previous studies, elevated fibrinogen levels might be responsible for this phenomenon [45,46]. Supporting this, we found significant positive correlations between fibrinogen levels and both lag time (r = 0.582, *p* = 0.021) and time to peak (r = 0.215, *p* = 0.035).

When analyzing clinical and tumor-related parameters at enrollment, we identified active smoking and lower hemoglobin levels among clinical variables, and Ki67 among tumor-related markers. Interestingly, no effect of specific antitumor therapies was observed, as reported in a recent comprehensive analysis of thrombosis associated with breast cancer, where anthracyclines, platinum-based therapies, hormonal treatments like tamoxifen, and targeted therapies including CDK4/6 inhibitors were linked to an increased risk of VTE [15]. Specifically, taxanes are known to be particularly thrombogenic. The primary mechanism is believed to involve direct endothelial damage and a pro-inflammatory response [9]. Contrastingly, tamoxifen induces a procoagulant state by increasing the production of several procoagulant factors, such as fibrinogen and factor VIII, while also decreasing the production of natural anticoagulants like antithrombin [47].

Ki-67 is an important prognostic biomarker in breast cancer, especially for assessing tumor growth and guiding treatment decisions, such as reducing treatment intensity based on cancer aggressiveness [48], with higher levels associated with worse outcomes [49]. However, its link to VTE in breast cancer has been less thoroughly studied. Only one study to date has identified a predictive value for this marker in a retrospective cohort of patients with ovarian cancer, which was incorporated into a nomogram designed to predict VTE, along with hormone receptor status (i.e., PR and ER), and age [50]. Interestingly, a recent study observed that within the tumor microenvironment of breast cancer, the expression of coagulation factors by fibroblasts correlates with aggressive cancer characteristics, such as high Ki-67, high grade, ER-negative status, and HER2-positive status [51]. Even if no direct mechanism is associated with Ki-67 and thrombosis, the connection is not due to a single factor but rather a complex interplay of tumor aggressiveness, systemic inflammation, and direct endothelial damage. High Ki-67 expression directly indicates a highly proliferative and aggressive tumor. These rapidly growing tumor cells often overexpress TF, a potent initiator of the coagulation cascade. The more aggressive the tumor (indicated by a high Ki-67), the greater its capacity to express and shed TF-expressing microparticles and circulating tumor cells into the bloodstream, creating a state of chronic hypercoagulability [52]. Additionally, aggressive tumors release pro-inflammatory cytokines (e.g., TNF-α, IL-1β), which activate host cells. This activation leads to increased expression of TF and other procoagulant proteins, creating a systemic inflammatory environment that favors coagulation. In addition, Ki-67-positive tumor cells have been correlated with neo-angiogenesis, which can lead to leaky and dysfunctional vasculature [53]. Lastly, the interaction between tumor cells and the host’s endothelial cells can cause direct damage, disrupting the vessel lining’s natural anticoagulant properties. Such damage exposes the underlying subendothelial matrix, resulting in platelet adhesion and activation. Furthermore, tumor-derived microparticles that contribute to hypercoagulability also directly affect endothelial cell function, promoting a pro-thrombotic environment and aiding in blood clot formation [54].

Having identified a series of significant laboratory, clinical, and tumor-related parameters, we submitted all these variables to a deeper statistical analysis aiming to generate a RAM for VTE. These analyses ultimately identified fibrinogen and Ki-67 as the most important independent predictors. A model was then generated by combining these two parameters, demonstrating high accuracy with a c-statistic of 0.78 and the ability to identify patients at twice the risk of VTE (SHR 3.59, *p* = 0.018). Other published RAMs (i.e., KRS, CATS nomogram, PROTECHT, COMPASS-CAT, HYPERCAN-lung scores) were unable to estimate the risk of VTE within our cohort accurately. This may be due to the different proportions of breast cancer patients included in these models. KRS included 35% of breast cancer patients in both its derivation and validation cohorts [25], while the CATS nomogram included only 16%, which may limit its predictive accuracy for this type of cancer group [26]. The COMPASS-CAT score, which comprises 61% of breast cancer patients in its derivation cohort and incorporates treatments specifically targeting breast cancer, also failed to correctly classify our cohort’s VTE risk when applying the validated cut-off value of 7 points [24]. Notably, a higher cut-off for the COMPASS-CAT score (≥11 points) has been proposed by Rupa-Matysek et al. [55]. This cut-off was established through ROC curve analysis, resulting in a c-statistic of 0.891, indicating stronger discrimination among lung cancer patients. Interestingly, the 11-point cut-off showed significant stratification for VTE in our breast cancer cohort, though it was narrowly within the limit of statistical significance (*p* = 0.045). The finding of a 0% VTE incidence in the low-risk group is a powerful and promising data point for identifying patients who might safely avoid thromboprophylaxis, suggesting the need to validate the COMPASS-CAT 11-point cut-off in future studies. Interestingly, our model shows that it outperforms COMPASS-CAT11 in terms of statistical significance and reliability. The Kaplan–Meier *p*-value for our score is 0.018, which is more significant than the 0.045 for COMPASS-CAT, indicating better risk discrimination and stratification. Furthermore, our score has an AUC of 0.782, demonstrating strong predictive ability, while the AUC for COMPASS-CAT is 0.616, which is not statistically significant (*p* = 0.151). This statistical performance also indicates a better clinical balance. Our score’s number needed to treat (NNT) is 8, meaning fewer patients need treatment to prevent one VTE event compared to the NNT of 10 for COMPASS-CAT. This increased efficiency reduces patient exposure to anticoagulation risks, such as bleeding, while maintaining therapeutic benefit.

VTE models specifically focused on breast cancer patients have been developed, including one recently published retrospective study that collected data from breast cancer patients undergoing chemotherapy [56]. This study developed a nomogram incorporating D-dimer, FDP, hemoglobin levels, leukocyte counts, age, surgical history, carcinoembryonic antigen levels, and cancer stage, yielding a c-statistic of 0.77. However, it was designed as a single-center retrospective cohort study. Another study developed a model to assess the post-operative risk in breast cancer patients, including variables such as age, BMI, cardiovascular comorbidities, neoadjuvant chemotherapy, surgical treatments, hospital length of stay, D-dimer, and homocysteine [57]. Yet, the model is designed for hospitalized patients and those undergoing surgery, so it differs from ours.

An additional key finding of our study is that the occurrence of VTE had a significant impact on the overall mortality rate during the follow-up period. This confirms data from Chew et al., who reported, through multivariate models, that VTE was a significant predictor of decreased 2-year survival (HR, 2.3; 95% CI, 2.1–2.6) [58]. Similarly, a population-based cohort study conducted in Denmark assessed a cohort of 4288 patients with breast cancer and VTE, alongside a control group of 12,847 breast cancer patients without VTE [59]. Among those diagnosed with VTE post-breast cancer, the one-year mortality risk was found to be 32%, escalating to 58% at the five-year mark. In contrast, the control group exhibited a one-year mortality risk of 7% and a five-year risk of 28%. This finding indicates that VTE is a serious complication even with advances in cancer treatment, highlighting the need for better models to identify at-risk patients and for careful monitoring and management of VTE in cancer patients. Therefore, the significant global prevalence of breast cancer and the additional metastatic status contribute to a considerable number of patients at risk for VTE complications and associated morbidity and mortality. This increased mortality also translates into substantial healthcare burdens, including prolonged hospital stays and increased resource utilization, further highlighting the urgency of effective VTE prevention and management.

Our study acknowledges several limitations. First, the relatively small sample size of 189 patients, all Italians, may restrict the generalizability of the findings, and broader demographic representation would be crucial to improve the generalizability of these findings. However, this limitation is partially counterbalanced by the prospective, multicenter, and observational design of our cohort. This approach allowed for careful and standardized data collection, lowering recall bias and creating a clear timeline linking clinical events and outcomes. Although the sample size limits broad application, it provides valuable real-world insights into the clinical course and outcomes within our specific patient group. In addition, longitudinal assessment of the collected data would be a valuable next step, providing a deeper understanding of VTE risk throughout treatment. This is a crucial area we intend to explore in future work. Second, Ki67 assessment can be subjective and vary across laboratories, highlighting the need for standardized methods for accurate evaluation. Finally, future research should aim to validate these findings in large, multicenter cohorts to strengthen the robustness of the results.

## 5. Conclusions

In conclusion, the results of our study highlight the significant incidence of VTE in patients with metastatic breast cancer who are undergoing chemotherapy. These findings possess the potential to enhance risk stratification and inform personalized treatment strategies for breast cancer patients, ultimately leading to improved patient outcomes. By identifying individuals who are at elevated risk of VTE, healthcare professionals can implement targeted prophylactic measures and optimize cancer treatment plans to mitigate the effects of thrombotic complications. This emphasizes the importance of a personalized approach to assessing thrombosis risk, which should take into account each patient’s specific treatment regimen and pre-existing conditions, thereby striving to achieve the highest possible health-related quality of life while maximizing survival rates.

## Figures and Tables

**Table 1:** General characteristics of the study population according to VTE development within one year and VTE-free patients.

	Overall Cohort (*n* = 189)	VTE-Free (*n* = 175)	1 Year-VTE (*n* = 14)	*p*-Value
Female sex, *n* (%)	185 (98)	172 (98)	13 (93)	0.271
Age, years, mean (SD)	60 (12.5)	60 (12.6)	59 (10.7)	0.725
BMI, kg/m^2^, mean (SD)	26 (5.4)	26 (5.4)	26 (5.3)	0.910
BMI ≥ 35 kg/m^2^, *n* (%)	12 (6)	11 (6)	1 (7)	0.629
ECOG, *n* (%)				
• 0	114 (60)	110 (63)	4 (29)	0.131
• 1	50 (27)	43 (25)	6 (43)	
• 2	16 (9)	15 (9)	1 (7)	
Smoking, *n* (%)				
• Active	15 (8)	12 (7)	3 (21)	0.020
• Previous	21 (11)	17 (8)	3 (21)	
Comorbidities ≥ 1, *n* (%)	102 (54)	96 (55)	6 (43)	0.619
• Diabetes	18 (10)	17 (10)	1 (7)	0.701
• Hypertension	64 (34)	59 (34)	5 (36)	0.512
• Dyslipidemia	12 (6)	12 (7)	0 (0)	-
• Cardiopathy	1 (0.5)	1 (0.6)	0 (0)	-
Central venous catheter, *n* (%)	24 (13)	23 (13)	1 (7)	0.641
Antithrombotic therapy, *n* (%)				
• Antiplatelet drugs	5 (3)	4 (2)	1 (7)	0.264
• Anticoagulants *	4 (2)	4 (2)	0 (0)	-
Histological subtypes, *n* (%)				
• Ductal	156 (83)	145 (83)	11 (79)	0.852
• Lobular	16 (9)	14 (8)	2 (14)	
• Not classified	17 (9)	16 (9)	1 (7)	
Molecular subtype, *n* (%)				
• Luminal A	27 (14)	25 (14)	2 (14)	0.957
• Luminal B	69 (37)	64 (37)	5 (36)	
• Luminal B HER2-positive	50 (27)	46(26)	4 (29)	
• HER 2-positive	17 (9)	16 (9)	1 (7)	
• Triple-negative	21 (11)	19 (11)	2 (14)	
• Not classified	5 (3)	3 (2)	2 (14)	
Chemotherapy, *n* (%)				
• Anthracycline	37 (20)	34 (19)	3 (21)	0.680
• Taxane	118 (62)	109 (62)	9 (64)	
• Anthracycline/taxane	15 (8)	15 (9)	0 (0)	
• Other	16 (9)	14 (8)	2 (14)	
Immunotherapy first-line, *n* (%)	81 (43)	72 (41)	8 (57)	0.219
Endocrine therapy, *n* (%)				
• Tamoxifen	100 (53)	93 (53)	7 (50)	0.473
• LHRH antagonists	21 (11)	19 (11)	2 (14)	
• Aromatase inhibitors	80 (42)	75 (43)	5 (36)	
Blood count, median (IQR)				
• Leukocytes, 109/L	6.75 (5.22–8.39)	6.70 (5.10–8.30)	6.50 (5.50–8.94)	0.949
• Hemoglobin, g/dL	13.0 (12.0–13.8)	13.0 (12.0–13.8)	12.5 (11.3–13.4)	0.112
• Hematocrit, %	39.3 (36.4–42.1)	39.1 (36.5–42.1)	39 (34.1–40.3)	0.240
• Platelets, 109/L	257 (209–307)	256 (209–305)	271 (222–356)	0.456
Blood count KRS cut-off, *n* (%)				
• Leukocyte >11 × 109/L	15 (8)	14 (8)	1 (7)	0.995
• Hemoglobin, <10 g/dL	7 (4)	5 (3)	2 (14)	0.093
• Platelets, >350 × 109/L	24 (13)	20 (11)	4 (29)	0.083

* At prophylactic dose. Categorical data are shown as number (*n*) and percentage. Continuous variables are presented as median with interquartile range (IQR) or mean with standard deviation (SD). VTE: venous thromboembolism; BMI: body mass index; ECOG: Eastern Cooperative Oncology Group performance status; HER2: Human Epidermal Growth Factor Receptor 2; LHRH: luteinizing hormone-releasing hormone; KRS: Khorana risk score.

**Table 2:** Univariable and multivariable analysis for 12-month VTE.

Variables	Univariable			Multivariable		
Clinical and Tumor Characteristics	SHR	95% CI	*p*-Value	SHR	95% CI	*p*-Value
Age, years	0.991	0.957–1.028	0.635			
BMI kg/m^2^	0.996	0.913–1.088	0.933			
Leukocytes, ×10^9^/L	0.972	0.809–1.168	0.765			
Hemoglobin, g/dL	0.786	0.629–0.983	0.034	0.787	0.585–1.060	0.114
Platelets, ×10^9^/L	1.003	0.997–1.009	0.334			
Comorbidities ≥ 1	1.165	0.405–3.347	0.777			
Active smoking	3.603	0.909–4.284	0.068			
Histological subtypes						
• Ductal (vs. lobular)	1.979	0.187–2.897	0.570			
Ki67 %	1.021	1.000–1.043	0.049	1.055	1.009–1.103	0.017
Ki67 positivity	1.537	0.363–6.506	0.559			
Estrogen receptor	0.998	0.984–1.011	0.730			
Estrogen receptor positivity	1.063	0.293–3.860	0.926			
Progesterone receptor	0.992	0.976–1.008	0.329			
Progesterone receptor positivity	0.764	0.250–2.331	0.636			
HER2 status	1.517	0.534–4.311	0.434			
ECOG ≥ 2	1.755	0.398–7.743	0.458			
Molecular subtype						
• Triple-negative (vs. other)	1.336	0.290–6.147	0.710			
Central venous catheter	1.222	0.226–6.609	0.815			
Chemotherapy						
• Anthracycline-based	0.724	0.201–2.607	0.621			
• Taxane-based	0.684	0.230–2.034	0.495			
• Antracycline/taxane	-	-	-			
Immunotherapy first line	1.672	0.581–4.815	0.341			
Endocrine therapy						
• Tamoxifen	0.834	0.296–2.355	0.732			
• LHRH antagonists	1.249	0.292–5.349	0.765			
• Aromatase inhibitors	0.722	0.245–2.128	0.555			
Biomarkers						
F1 + 2, pmol/L	1.001	0.999–1.002	0.294			
D-dimer, µg/mL	1.238	1.026–1.495	0.026	0.991	0.629–1.560	0.968
Fibrinogen, mg/dL	1.007	1.003–1.010	<0.001	1.008	1.001–1.017	0.048
FVIII, %	1.007	1.001–1.014	0.028	1.015	1.001–1.030	0.034
TG lag time, min	1.438	1.112–1.858	0.006	0.474	0.098–2.278	0.351
TG peak, nM	1.001	0.998–1.004	0.250			
TG ttP, min	1.716	1.266–2.327	0.020	1.484	0.933–5.005	0.156
TG ETP, nM*min	1.001	1.000–1.002	0.034	1.000	0.998–1.002	0.980

Fine and Gray (FGM) univariable Cox proportional hazard model analysis evaluating 12-month VTE. The FGM multivariable model considered the significant (*p* < 0.05) variable obtained from the univariable analysis. SHR: subdistribution hazard ratio; CI: confidence interval; BMI: body mass index; ECOG: Eastern Cooperative Oncology Group performance status; HER2: Human Epidermal Growth Factor Receptor 2; LHRH: luteinizing hormone-releasing hormone; FVIII: factor VIII; F1 + 2: prothrombin fragment 1 + 2; TG: thrombin generation; ttp: time to peak; ETP: endogenous thrombin potential.

**Table 3:** Univariable and multivariable analysis for 1-year mortality.

Variables	Univariable			Multivariable		
Clinical and Tumor Characteristics	HR	95% CI	*p*-Value	HR	95% CI	*p*-Value
Age, years	1.007	0.976–1.038	0.665			
BMI kg/m^2^	0.950	0.873–1.034	0.237			
Leukocytes, ×10^9^/L	1.145	1.011–1.296	0.033	1.150	1.000–1.322	0.050
Hemoglobin, g/dL	1.030	0.787–1.347	0.831			
Platelets, ×10^9^/L	1.002	0.997–1.007	0.341			
Cardiovascular risk factors ≥ 1	0.670	0.304–1.476	0.320			
Active smoking	1.074	0.240–4.799	0.926			
Histological subtypes						
• Ductal (vs. Lobular)	1.118	0.263–4.753	0.880			
Ki67 %	1.009	0.991–1.028	0.317			
Ki67 positivity	1.215	0.415–3.555	0.722			
Estrogen receptor %	0.990	0.980–1.000	0.052			
Estrogen receptor positivity	0.575	0.308–1.072	0.082			
Progesterone receptor %	0.979	0.962–0.996	0.016			
Progesterone receptor positivity	0.541	0.295–0.992	0.047			
HER2 status	0.660	0.272–1.605	0.360			
ECOG ≥ 2	4.616	1.830–11.64	0.001			
Molecular subtype						
• Triple-negative (vs. other)	3.318	1.848–5.958	<0.001	3.988	2.059–7.724	<0.001
Central venous catheter	0.643	0.231–1.786	0.397			
Chemotherapy						
• Anthracycline-based	0.550	0.154–1.968	0.358			
• Taxane-based	0.809	0.160–4.094	0.798			
• Antracycline/taxane	0.407	0.185–0.893	0.025			
Immunotherapy first line	0.284	0.107–0.753	0.011			
Endocrine therapy						
• Tamoxifen	0.249	0.117–0.530	<0.001			
• LHRH antagonists	0.104	0.002–4.400	0.236			
• Aromatase inhibitors	0.160	0.048–0.536	0.003			
Biomarkers						
F1 + 2, pmol/L	1.000	0.999–1.002	0.207			
D-dimer, µg/mL	1.257	1.060–1.491	0.008			
Fibrinogen, mg/dL	1.003	1.001–1.006	0.009			
FVIII, %	1.008	1.004–1.014	<0.001	1.010	1.005–1.016	<0.001
TG lag time, min	1.160	0.865–1.556	0.323			
TG peak, nM	1.001	0.999–1.003	0.213			
TG ttP, min	1.033	0.821–1.299	0.780			
TG ETP, nM*min	1.000	0.999–1.001	0.341			

Univariable Cox proportional hazard model analysis evaluating 12-month mortality. The multivariable analysis considered the significant variables (*p* < 0.05) identified in the univariable analysis. HR: hazard ratio; CI: confidential interval; BMI: body mass index; ECOG: Eastern Cooperative Oncology Group performance status; HER2: Human Epidermal Growth Factor Receptor 2; LHRH: luteinizing hormone-releasing hormone; FVIII: factor VIII; F1 + 2: prothrombin fragment 1 + 2; TG: thrombin generation; ttp: time to peak; ETP: endogenous thrombin potential.

## Data Availability

The data presented in this study are available on request from the corresponding author.

## Supplementary Materials

The following supporting information can be downloaded at https://www.mdpi.com/article/10.3390/cancers17162712/s1, Table S1. Thrombogenic mechanisms of chemotherapy associated with VTE in breast cancer. Figure S1: Heat map of the correlation of biomarkers (A) in patients without venous thromboembolism (VTE), (B) in patients with 12-month VTE; Figure S2: Calibration curve of the model; Figure S3: Kaplan–Meier of published RAMS (A) Khorana risk score (KRS) at 3 points, (B) KRS at 2 points, (C) Vienna CATS nomogram, (D) HYPERCAN-VTE, (E) COMPASS-CAT at 7 points cut-off, (F) COMPASS-CAT at 11 points cut-off. References [60,61,62,63,64,65,66,67] are cited in the Supplementary Materials.

## Appendix A

**Collaborators:** The members of the HYPERCAN study (by centers, all in Italy) are the following: Coordinating Center: Immunohematology and Transfusion Medicine, Hospital Papa Giovanni XXIII, Bergamo: Falanga Anna, Marchetti Marina, Gomez-Rosas Patricia, Testa Maria, Motta Alessia, Tartari Carmen Julia, Russo Laura, Bolognini Silvia, Ticozzi Chiara, Debora Romeo, Schieppati Francesca, Luca Barcella. Participants: Oncology Unit, IRCCS Humanitas Research Hospital, Rozzano Milan: Masci Giovanna, Santoro Armando. Oncology Unit, IRCCS National Cancer Institute, Milan: De Braud Filippo, Celio Luigi. Oncology Unit, Hospital Papa Giovanni XXIII, Bergamo: Tondini Carlo, Labianca Roberto. Oncology Unit, Hospital San Filippo Neri, Rome: Gasparini Giampietro, Sarmiento Roberta. Oncology Unit, Hospital Treviglio-Caravaggio, Treviglio: Petrelli Fausto. Medical oncology and Internal Medicine, University Vita-Salute San Raffaele, Milan: D’Alessio Andrea. Oncology Unit, IRCCS Cancer Institute Giovanni Paolo II, Bari: Giuliani Francesco.
