# Supplementary material for: Baseline Hemostatic Biomarker Assessment Identifies Breast Cancer Patients at High Risk for Venous Thromboembolism During Chemotherapy
Source: Cancers (Basel). 2025 Aug 20;17(16):2712. doi: 10.3390/cancers17162712 (PMC12384613; doi:10.3390/cancers17162712)
Supplement: Supplementary file 1 [file cancers-17-02712-s001.zip › Supplementary Table S1.pdf]

| Agent | Proposed Mechanism of Thrombogenesis | Rate of thrombosis in Breast cancer |
|---|---|---|
| **Cyclophosphamide** | Direct endothelial cell damage through cyclophosphamide metabolites such as acrolein.<br>Combined effect on coagulation and fibrinolysis. | 7-10% [1,2] |
| **Tamoxifen** | Estrogenic effects on the liver, altering the balance of pro- and anti-coagulant factors. | 1.2-2.3 %, when used alone [3]<br>8% in combination with cyclophosphamide [4] |
| **Platinum** | Induces endothelial cell apoptosis, releasing procoagulant microparticles. Increase von Willebrand factor (vWF) levels, increasing thrombin generation via tissue factor-independent pathways. | 1-18% [5] |
| **Fluorouracil (5-FU)** | Endothelial damage, potentially leading to microvascular injury.<br>Depletes protein C and increases thrombin activity. | 17.6% in combined schemes [6] |
| **Bevacizumab** | VEGF inhibition primes the endothelium for injury.<br>VEGF blockade reduces thrombus resolution and neovascularization.<br>Its effect on the vascular system can lead to both arterial and venous thrombotic events. | 7.3 % [7] |
| **Erythropoiesis-Stimulating Agents** | Increased red blood cell mass and blood viscosity. Raises the risk of thrombosis, particularly if hemoglobin targets are high. | 8.5% [8] |
| **Granulocyte Colony-Stimulating Factors (G-CSF)** | Activation of neutrophils and endothelial cells leads to an increase in hypercoagulability, evidenced by elevated levels of prothrombin fragment F1+2, thrombin-antithrombin complex, and D-dimer. | 1.9 -2.3 [9] |
| **Taxanes (e.g., Paclitaxel, Docetaxel)** | Direct endothelial damage and induction of a pro-inflammatory state. | 2.4-3.2% [10] |

1 Krüger-Genge A, Köhler S, Laube M, Haileka V, Lemm S, Majchrzak K, Kammerer S, Schulz C, Storsberg J, Pietzsch J, Küpper JH, Jung F. Anti-Cancer Prodrug Cyclophosphamide Exerts Thrombogenic Effects on Human Venous Endothelial Cells Independent of CYP450 Activation-Relevance to Thrombosis. Cells. 2023 Jul 29;12(15):1965. doi: 10.3390/cells12151965. PMID: 37566045; PMCID: PMC10416884.

2 Levine MN, Gent M, Hirsh J, Arnold A, Goodyear MD, Hryniuk W, De Pauw S. The thrombogenic effect of anticancer drug therapy in women with stage II breast cancer. N Engl J Med. 1988 Feb 18;318(7):404-7. doi: 10.1056/NEJM198802183180703. PMID: 3340118

3 Hernandez RK, Sørensen HT, Pedersen L, Jacobsen J, Lash TL. Tamoxifen treatment and risk of deep venous thrombosis and pulmonary embolism: a Danish population-based cohort study. Cancer. 2009 Oct 1;115(19):4442-9. doi: 10.1002/cncr.24508. PMID: 19569248.]

4 Saphner T, Tormey DC, Gray R. Venous and arterial thrombosis in patients who received adjuvant therapy for breast cancer. J Clin Oncol. 1991 Feb;9(2):286-94. doi: 10.1200/JCO.1991.9.2.286. PMID: 1988575.

5 Seng S, Liu Z, Chiu SK, Proverbs-Singh T, Sonpavde G, Choueiri TK, Tsao CK, Yu M, Hahn NM, Oh WK, Galsky MD. Risk of venous thromboembolism in patients with cancer treated with Cisplatin: a systematic review and meta-analysis. J Clin Oncol. 2012 Dec 10;30(35):4416-26. doi: 10.1200/JCO.2012.42.4358. Epub 2012 Nov 13. PMID: 23150697.

6 Goodnough LT, Saito H, Manni A, Jones PK, Pearson OH. Increased incidence of thromboembolism in stage IV breast cancer patients treated with a five-drug chemotherapy regimen. A study of 159 patients. Cancer. 1984 Oct 1;54(7):1264-8. doi: 10.1002/1097-0142(19841001)54:7<1264::aid-cncr2820540706>3.0.co;2-r. PMID: 6547874.]

7 Nalluri SR, Chu D, Keresztes R, Zhu X, Wu S. Risk of venous thromboembolism with the angiogenesis inhibitor bevacizumab in cancer patients: a meta-analysis. JAMA. 2008 Nov 19;300(19):2277-85. doi: 10.1001/jama.2008.656. PMID: 19017914.

8 Zhan P, Wang Q, Qian Q, Yu LK. Risk of venous thromboembolism with the erythropoiesis-stimulating agents (ESAs) for the treatment of cancer-associated anemia: a meta-analysis of randomized control trials. Chin Clin Oncol. 2012 Dec;1(2):19. doi: 10.3978/j.issn.2304-3865.2012.12.10. PMID: 25841397.

9 Du XL, Zhang Y, Hardy D. Associations between hematopoietic growth factors and risks of venous thromboembolism, stroke, ischemic heart disease and myelodysplastic syndrome: findings from a large population-based cohort of women with breast cancer. Cancer Causes Control. 2016 May;27(5):695-707. doi: 10.1007/s10552-016-0742-5. Epub 2016 Apr 8. PMID: 27059219.

10 Mackey JR, Ramos-Vazquez M, Lipatov O, McCarthy N, Krasnozhon D, Semiglazov V, Manikhas A, Gelmon KA, Konecny GE, Webster M, Hegg R, Verma S, Gorbunova V, Abi Gerges D, Thireau F, Fung H, Simms L, Buyse M, Ibrahim A, Martin M. Primary results of ROSE/TRIO-12, a randomized placebo-controlled phase III trial evaluating the addition of ramucirumab to first-line docetaxel chemotherapy in metastatic breast cancer. J Clin Oncol. 2015 Jan 10;33(2):141-8. doi: 10.1200/JCO.2014.57.1513. Epub 2014 Sep 2. PMID: 25185099.
